# Colossal Permittivity and Low Dielectric Loss of Thermal Oxidation Single-Crystalline Si Wafers

**DOI:** 10.3390/ma12071102

**Published:** 2019-04-03

**Authors:** Yalong Sun, Di Wu, Kai Liu, Fengang Zheng

**Affiliations:** College of Physical Science and Technology, School of Optoelectronic Science and Engineering and Technology and Jiangsu Key Laboratory of Thin Films, Soochow University, Suzhou 215006, China; 20164208051@stu.suda.edu.cn (Y.S.); 20154208020@stu.suda.edu.cn (K.L.)

**Keywords:** colossal permittivity, single-crystalline silicon plate, thermal oxidation

## Abstract

In this work, thin SiO_2_ insulating layers were generated on the top and bottom surfaces of single-crystalline silicon plates (n type) by thermal oxidation to obtain an insulator/semiconductor/insulator (ISI) multilayer structure. X-ray diffraction (XRD) pattern and scanning electron microscope (SEM) pictures implied that all of the synthesized SiO_2_ layers were amorphous. By controlling the thermal oxidation times, we obtained SiO_2_ layers with various thicknesses. The dielectric properties of silicon plates with different thicknesses of SiO_2_ layers (different thermal oxidation times) were measured. The dielectric properties of all of the single-crystalline silicon plates improved greatly after thermal oxidation. The dielectric constant of the silicon plates with SiO_2_ layers was approximately 10^4^, which was approximately three orders more than that of the intrinsic single-crystalline silicon plate (11.9). Furthermore, both high permittivity and low dielectric loss (0.02) were simultaneously achieved in the single-crystalline silicon plates after thermal oxidation (ISI structure).

## 1. Introduction

High dielectric constant (colossal permittivity, CP) materials are indispensable for producing modern advanced microelectronics and high density storage systems. Thus, much research has focused on high dielectric constant materials. High performance dielectric constant materials must have the following three conditions: (1) stable dielectric properties in a wide range of frequencies and temperatures, (2) larger dielectric constants (*ε_r_* > 10^3^), and (3) relatively small loss (tan δ < 0.1). However, a common problem in the study of materials with high dielectric constants is that the dielectric loss may become very large when the dielectric constant is increased. To date, several mechanisms have been proposed to explain the CP in high dielectric materials (CP > 10^3^) [1,2,3,4,5,6,7,8,9,10,11], such as BaTiO_3_ ceramic oxides [9,10], CaCu_3_Ti_4_O_12_ (CCTO [2,5,6,7,8]), NiO [3], La_2x_Sr_x_NiO_4_ [4] (x = 1/3 or 1/8), and doped TiO_2_ [5,11]. There are several CP mechanisms, including the barrier layer capacitor mechanism [1,12], the nanoscale atomic dislocation mechanism [13,14], Mott’s relation obey the variable range hopping (VRH) mechanism [15], and the electric dipole pinning effect [16,17]. If there are a large number of defects in a polycrystalline material, the space charges caused by the defects could be bounded near the interface among the grains. Due to thermal excitation, these space charges can jump from one place to another, forming a large quantity of carriers (similar to free charge), which is also a mechanism to obtain a high dielectric constant. If these carriers are localized near the lattice defects, a larger dielectric constant and a smaller dielectric loss can be obtained simultaneously. Unfortunately, under the action of the applied electric field, these jumping carriers usually participate in the conduction process of the external circuit, resulting in large leakage related losses [18,19]. 

In the inner region of a single-crystalline material, there is no any grain interface for bounding the space charges. Therefore, it is usually considered impossible that both a high dielectric constant and a low dielectric loss can be simultaneously achieved by adding the number of carriers in a single-crystalline material. However, it is possible that, on the surface of a single-crystalline material, an insulating layer can be prepared artificially to isolate the space charge. If so, under an applied electric field, internal polarization can form because positive and negative space charges are separated and accumulated at the top or the bottom surface, respectively. In other words, the inner mobile carriers are bound under the top or the bottom surface due to the insulating layers. Then, a low leakage related loss with a large polarization due to a large amount of space charges can occur, namely, a high dielectric constant and low dielectric loss can be obtained simultaneously.

In our recent work [20], we successfully lowered the dielectric loss of a TiO_2_ ceramic with richer oxygen vacancies by introducing insulating layers on both the top and the bottom surfaces. We proposed a route to realize high performance CP by creating multilayer structures of the insulator/semiconductor/insulator (ISI). In this paper, a SiO_2_/Si/SiO_2_ structure in single-crystal semiconductor Si wafers by thermal oxidation was obtained by generating an amorphous SiO_2_ film on the top and the bottom surfaces, and the microstructure and dielectric properties of the SiO_2_/Si/SiO_2_ structures were studied in detail. 

## 2. Materials and Methods

The raw material used in this work was n-type Si<100>. The Si wafers (1 × 1 cm^2^) were cleaned sequentially with 1% HF (mass fraction) and alcohol in an ultrasonic bath for 10 min each and dried after each step under an N_2_ stream. The Si wafers were put into a tubular furnace for thermal oxidation. The tubular furnace temperature was 1100 °C, and the flow of O_2_ atmosphere was 10.0 sccm during the thermal oxidation process. After thermal oxidation, the Si wafers were removed from the tubular furnace and cooled naturally to room temperature. The Si wafers underwent thermal oxidation at 1100 °C (with O_2_) for 3 min, 5 min, 10 min, 20 min, 40 min, and 1 h, respectively. Samples of the thermal oxidation Si wafers with different Si wafer resistivity and different Si wafer thickness were prepared using the control variable method. Ag electrodes (3.14 mm^2^) were prepared on both sides of the thermal oxidation Si wafers by magnetron sputtering in an Ar atmosphere (40.0 sccm) for 5 min. The sputtering power was 40 W, and the pressure of the Ar atmosphere was 1.0 Pa during the sputtering process.

The crystal structures of the Si wafers were evaluated by measuring the XRD patterns (RIGAKU D/MAX 3C, The Woodlands, TX, USA) recorded using Ni-filtered CuKα radiation (λ = 1.540598 Å, scanning range from 20° to 80° and step length of 6°/min). The surface morphologies of the Si wafers were investigated using SEM (Hitachi S-570, Tokyo, Japan). Dielectric properties were measured by the impedance analyzer (HP4294A). X-ray photoelectron spectroscopy (XPS, Escalab 250 XI, Thermo Fisher Scientific, Waltham, MA, USA) was also collected from the thermal oxidation Si wafer samples to assess the valance states of the Si ions and roughly evaluate the oxygen content.

## 3. Results and Discussion

### 3.1. Microstructure Analysis of the Single-Crystal Semiconductor Si Plates after Thermal Oxidation

**Figure 1:**
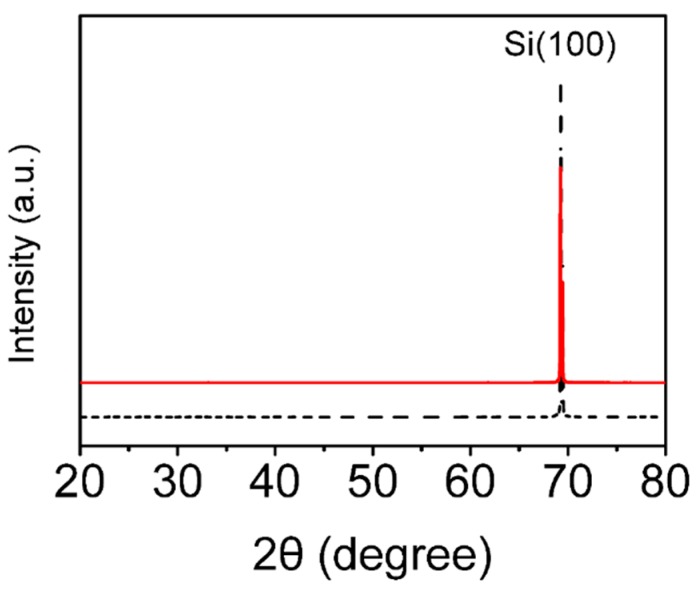
XRD patterns of a single-crystalline silicon plate (thickness 0.7 mm, resistivity 0.001 Ω∙cm). Raw single-crystalline silicon wafer (black dash line) and after thermal oxidation for 3 min (red solid line).

The high temperature of thermal oxidation process is usually 900–1200 °C [21] to form a layer of SiO_2_ thin films on the Si surface. It mainly includes three kinds of different methods: dry oxygen oxidation, wet oxidation, and oxygen water vapor oxidation [22,23]. In this paper, the dry oxygen oxidation method was used to thermally oxidize the single-crystal semiconductor Si plates. Figure 1 shows the XRD pattern of a single-crystalline silicon plate before and after thermal oxidation for 3 min. The XRD pattern shows there was only a peak at the Si<100> crystalline phase, which suggests no new crystalline phase was generated during the thermal oxidation process. The surface morphologies of the above two samples were observed by SEM, as shown in Figure S1 in Supplementary Files. There was no grain or grain boundary on the surface of the Si plate, even at a magnification of 90,000 times, which was consistent with the measured XRD results, indicating that the SiO_2_ film generated by thermal oxidation was amorphous. An amorphous SiO_2_ thin film [24,25] is a tetrahedral network structure where the oxygen atoms between two tetrahedrons are called bridge bonded oxygen atoms, and those only associated with one tetrahedron are called non-bridge bonded oxygen atoms.

### 3.2. XPS Analysis and SiO_2_ Film Thickness Measurement of the Single-Crystalline Silicon Plates after Thermal Oxidation

**Figure 2:**
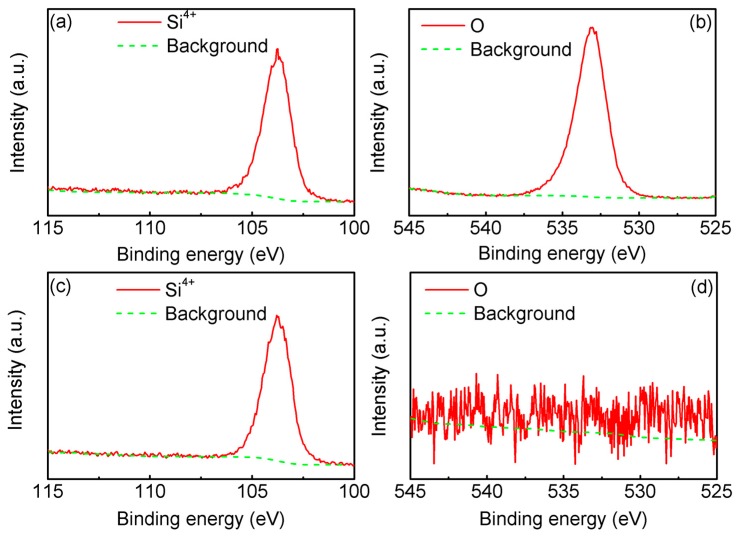
X-ray photoelectron spectroscopy (XPS) data of Si 2p and O 1s electrons for the single-crystalline silicon plates after thermal oxidation (3 min) before etching using the Ar ion process (**a**,**b**) and after etching using the Ar ion process (**c**,**d**). (**a**,**c**) and (**b**,**d**) are the surface XPS data of Si 2p and O 1s, respectively.

To determine the thickness of the SiO_2_ layer and check the valance state of the Si and O elements in the thermally oxidized Si plates, we measured the XPS data of both the surface and the inner region using the Ar ion etching process. The results are shown in Figure 2.

Figure 2a,b shows the elements (Si and O) distribution and valence states on the surface of the thermal oxidized single-crystalline silicon plate before etching. The results show that the Si element had only a Si^4+^ valence state of a Si 2p orbital (103.8 eV), and the O element had only one peak (532.9 eV). The binding energy of the Si–O bond was consistent with that reported in the literature [26,27]. Since the thermal oxidation process of the single-crystalline silicon plates was carried out in an oxygenated atmosphere, Si^4+^ could not be reduced to low-valence ions at high temperature. According to the atomic content distribution obtained by XPS analysis, the contents of the Si and O elements on the surface of the Si sheet before etching were 30.83% and 57.9% (that of C was 11.27%, not shown here), respectively, which further demonstrated that a layer of SiO_2_ was generated on the surface of the Si plates after thermal oxidation. When Ar ion etching was used (the etching voltage was 2 kV, etching rate of the SiO_2_ was about 0.14 nm/s [28]) after the etching of 220 s, the surface O element was almost impossible to detect, as shown in Figure 2d. Here, the contents of the Si and O elements on the surface of Si plates were 89.25% and 0.5% (that of C was 10.25%, not shown here), respectively. 

**Figure 3:**
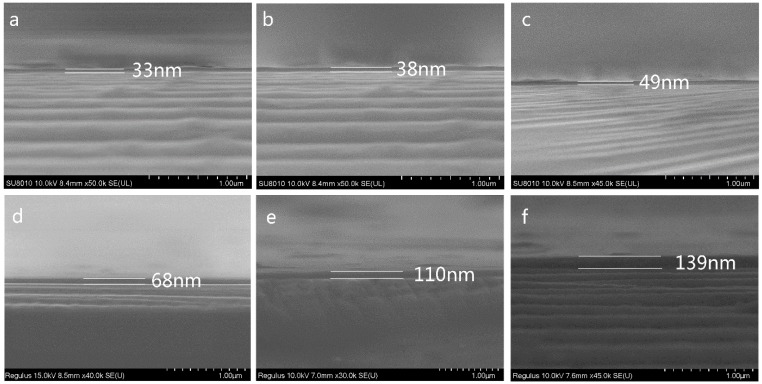
Cross-section SEM images of a single-crystalline silicon plate (thickness 0.7 mm, resistivity 0.001 Ω∙cm) at different thermal oxidation times (**a**) 3 min, (**b**) 5 min, (**c**) 10 min, (**d**) 20 min, (**e**) 40 min, and (**f**) 1 h.

The thickness of the etched SiO_2_ layers was derived from the product of the etching rate (0.14 nm/s) and etching time (220 s). Therefore, the thickness of the SiO_2_ obtained after 3 min of thermal oxidation was approximately 30.8 nm. Deal et al. [22] reported the relationship between the thickness of SiO_2_ layers generated by the thermal oxidation of Si plates and the thermal oxidation time, and the corresponding SiO_2_ thickness was similar to the results we measured. According to the thickness of the single-crystalline silicon plate after thermal oxidation for 3 min, we could evaluate the thickness of other thermal oxidation samples for 5 min, 10 min, 20 min, 40 min, and 1 h, respectively, i.e., 35 nm, 48 nm, 70 nm, 104 nm, and 131 nm. These evaluated SiO_2_ thicknesses were confirmed by the SEM cross-section pictures seen in Figure 3. Figure 3 shows the cross-section SEM images of a single-crystalline silicon plate (thickness 0.7 mm, resistivity 0.001 Ω∙cm) that underwent thermal oxidation for various times in the O_2_ atmosphere. The SiO_2_ layer thickness of the thermally oxidized silicon plates increased with the increasing thermal oxidation time. From Figure 3, we could evaluate the thickness of thermal oxidation samples for 3 min, 5 min, 10 min, 20 min, 40 min, and 1 h, respectively, i.e., 33 nm, 38 nm, 49 nm, 68 nm, 110 nm, and 139 nm.

### 3.3. Dielectric Properties at Room Temperature of the Single-Crystal Semiconductor Si Plates after Thermal Oxidation

**Figure 4:**
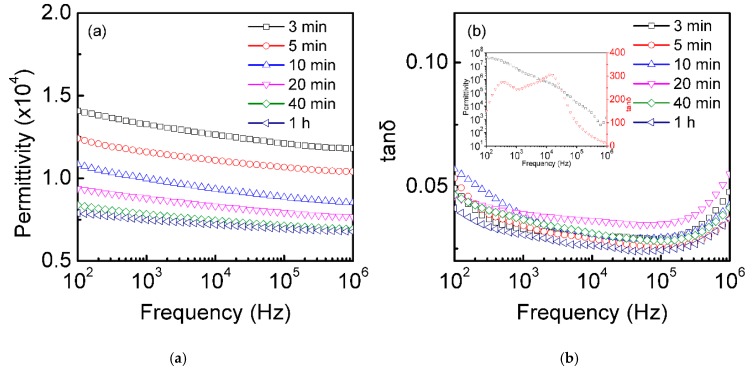
Frequency dependence of the dielectric properties at room temperature. Dielectric permittivity (**a**) and dielectric loss (**b**) of the single-crystalline silicon plates (0.7 mm) at different thermal oxidation times (3 min, 5 min, 10 min, 20 min, 40 min, and 1 h). The inset figure shows dielectric constant and dielectric loss of the raw single-crystalline silicon wafer (no thermal oxidation).

Figure 4 shows the dielectric permittivity and dielectric loss of the single-crystalline silicon plates (thickness 0.7 mm, resistivity 0.001 Ω·cm) that underwent thermal oxidation for various times in the O_2_ atmosphere. The dielectric permittivity of the thermally oxidized silicon plates decreased with the increasing thermal oxidation time. It was easy to understand that the thickness of the SiO_2_ generated at the top and the bottom of the silicon plates increased as the thermal oxidation time increased. According to our recent work [20], we found that the dielectric constant was inversely proportional to the thickness of the insulator in the ISI structure (seen in Figure S2 in Supplementary Files). The maximum dielectric permittivity (~1.4 × 10^4^) of the thermally oxidized silicon plates for 3 min was approximately two times higher than that of the thermally oxidized silicon plates for 1 h (~0.75 × 10^4^). The CP varied within 15% with frequency (100–10^6^ Hz) and independent of the temperature (100–250 K, seen in Figure S3 in Supplementary Files). The dielectric constant of all of the thermally oxidized silicon plates were enhanced more than that of the intrinsic single-crystal silicon plates (~11.9 [29]). The measured dielectric loss of the samples with different thermal oxidation times had no relationship with thermal time on the whole, which decreased with increased thermal oxidation time. The dielectric losses of the all samples were below 0.06 over most of the measured frequency range (10^2^–10^6^ Hz), and the minimum dielectric loss was 0.02 (at 10^4^–10^5^ Hz) for the thermally oxidized silicon plates for 1 h. Compared with that of the raw Si wafer (the inset of Figure 4b), the dielectric constant and dielectric loss of all of the thermally oxidized silicon plates were decreased greatly because the carriers were concentrated on the top and the bottom surfaces of the silicon plates due to the SiO_2_ generated by thermal oxidation, thus no large leakage current was generated, preventing large dielectric loss. 

**Figure 5:**
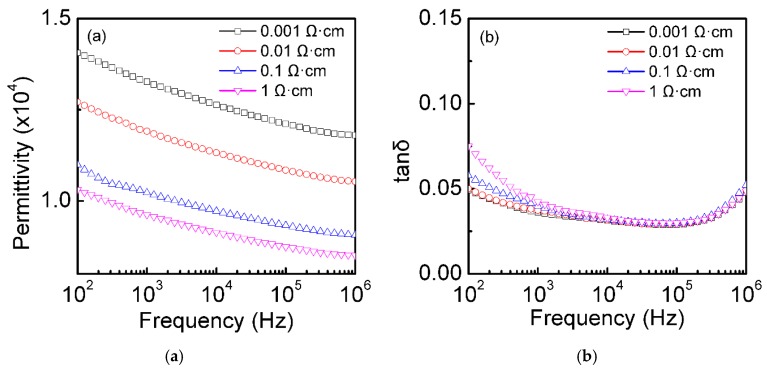
Frequency dependence of the dielectric properties at room temperature. Dielectric permittivity (**a**) and dielectric loss (**b**) of the single-crystalline silicon plates (0.7 mm) with different resistivity (0.001, 0.01, 0.1, and 1 Ω·cm) after thermal oxidation for 3 min.

To further study the effects of different resistivity on the dielectric properties of the silicon plates after oxidation, the dielectric permittivity and dielectric loss of the thermal oxidized Si plates (thickness 0.7 mm) with different resistivity at room temperature were assessed, as shown in Figure 5. The CP of the thermally oxidized silicon plates decreased with the increase of the resistivity. It was easy to understand that the larger the resistivity of the single-crystal Si plates was, the lower the internal carrier concentration was, which was very crucial to the polarization [20]. The maximum dielectric permittivity at 10^5^ Hz (~1.23 × 10^4^) was obtained when the resistivity of the silicon plates was 0.001 Ω·cm. The measured dielectric losses of the 0.001 Ω·cm resistivity sample were below 0.06 over most of the measured frequency range (10^2^–10^6^ Hz), and the minimum dielectric loss was 0.03 (at 2 × 10^4^–10^5^ Hz). The CP and dielectric loss changed slightly in the whole frequency range (10^2^–10^6^ Hz), indicating that the dielectric properties of the thermal oxidized single-crystal Si plates with different resistivity had better frequency stability.

**Figure 6:**
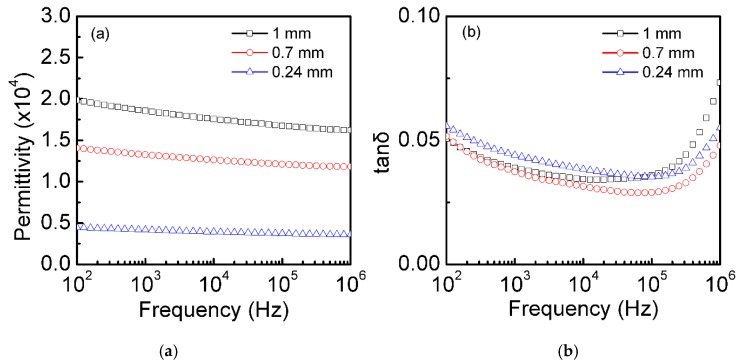
Frequency dependence of the dielectric properties at room temperature. Dielectric permittivity (**a**) and dielectric loss (**b**) of the single-crystalline silicon plates (0.001 Ω·cm) with different thicknesses (0.24, 0.7, and 1 mm) after thermal oxidation for 3 min.

According to the typical formula of a relative dielectric constant:

(1) $$\varepsilon_{r}=\frac{C\cdot d}{\varepsilon_{0}\cdot S},$$

here, ɛ_0_ was the absolute dielectric constant and S was the surface area of the top and bottom surfaces’ electrodes of the thermally oxidized single-crystalline silicon plates, C was the capacitance, and d was the thickness of the single-crystal silicon plates. Usually, C decreases with the increasing of d, and as a result, ɛ_r_ keeps a constant. It was interesting that the ɛ_r_ of the thermally oxidized Si plates was dependent on d (the thickness of the Si plates). Figure 6 shows the dielectric permittivity and the dielectric loss of the single-crystalline silicon plates (different thicknesses) thermally oxidized in an O_2_ atmosphere. The dielectric permittivity of the silicon plates increased with the increase of Si plate thickness, and the maximum dielectric permittivity at 100 Hz (~1.95 × 10^4^) obtained in the 1.0 mm thick Si plates was approximately 4.2 times higher than that (~0.46 × 10^4^) of the 0.24 mm thick Si plates, which was due to the measured C that had almost no large change with different thicknesses. This may have been attributed to the large carrier concentration that was plenty big enough for the polarization, which was not dependent on the single-crystalline silicon plates (different thickness). Furthermore, the CP was almost independent of the frequency (100–10^6^ Hz). The measured dielectric losses of the 1.0 mm sample were below 0.07 over most of the measured frequency range (10^2^–10^6^ Hz), and the minimum dielectric loss was 0.04 (at 10^4^–10^5^ Hz). Similarly, the CP and the dielectric loss changed slightly in the whole frequency range (10^2^–10^6^ Hz), indicating that the dielectric properties of the single-crystal Si plates with different thicknesses after thermal oxidation had better frequency stability.

## 4. Conclusions

In this study, thin SiO_2_ insulating layers were generated on the top and the bottom surfaces of single-crystalline silicon plates (n type) via thermal oxidation to obtain an ISI multilayer structure. The experiments were carried out under the conditions of controlling the thermal oxidation time, the resistivity of the Si plates, and the thickness of the Si plates, respectively. The maximum dielectric permittivity (10^4^) was obtained. Furthermore, the CP was almost independent of the frequency (100–10^6^ Hz). The measured dielectric losses of the sample were below 0.07 over most of the measured frequency range (10^2^–10^6^ Hz), and the minimum dielectric loss was 0.02 (at 10^4^–10^5^ Hz). The ISI structure obtained from the single-crystalline silicon plates after thermal oxidation had better dielectric characteristics, that is, high dielectric constant and low dielectric loss could be obtained at the same time.

## Supplementary Materials

The following are available online at https://www.mdpi.com/1996-1944/12/7/1102/s1, Figure S1: SEM image of a single-crystalline silicon plate (thickness 0.7 mm, resistivity 0.001 Ω∙cm). (**a**) Raw single-crystalline silicon wafer and (**b**) after thermal oxidation for 3 min, Figure S2: Schematic of colossal permittivity based on the thermal oxidation for 3 min single-crystalline silicon plates (thickness 0.7 mm, resistivity 0.001 Ω∙cm) in O_2_ atmosphere. (**a**) Insulator/semiconductor/insulator sandwich structure. (**b**) Sandwich structure applied by an external electrical field. (**c**) Dielectric constant and thickness of each part in the sandwich structure. ɛ_r1_ (ɛ_r2_) and d_1_ (d_2_) are dielectric constant and thickness of the insulator (semiconductor), respectively. The total thickness of the sandwich structure is labeled as d, which is the sum of d_1_ and d_2_ (d_2_ >> d_1_), Figure S3: Temperature dependence of the dielectric properties at difference frequency (500 Hz, 1 kHz, 10 kHz, 50 kHz and 100 kHz) were measured from 100 to 450 K. Dielectric permittivity (**a**) and dielectric loss (**b**) of the single-crystalline silicon plate (thickness 0.7 mm, resistivity 0.001 Ω∙cm) after thermal oxidation for 3 min.
