# Supplementary material for: Colossal Permittivity and Low Dielectric Loss of Thermal Oxidation Single-Crystalline Si Wafers
Source: Materials (Basel). 2019 Apr 3;12(7):1102. doi: 10.3390/ma12071102 (PMC6479920; doi:10.3390/ma12071102)
Supplement: Supplementary file 1 [file materials-12-01102-s001.pdf]

materials

Article

Colossal Permittivity and Low Dielectric Loss of Thermal Oxidation Single-Crystalline Si Wafers

Yalong Sun, Di Wu *, Kai Liu and Fengang Zheng *

College of Physical Science and Technology, School of Optoelectronic Science and Engineering and Technology and Jiangsu Key Laboratory of Thin Films, Soochow University, Suzhou 215006, China; 20164208051@stu.suda.edu.cn (Y.S.); 20154208020@stu.suda.edu.cn (K.L.)

* Correspondence: wudi@suda.edu.cn (D.W.); zhfg@suda.edu.cn (F.Z.)

Supplementary Material

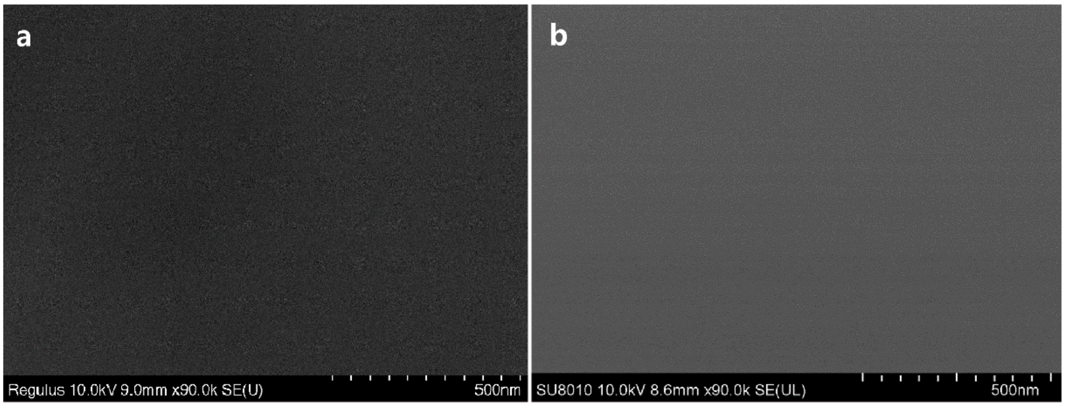

(**a**) (**b**)

Figure S1. SEM image of a single-crystalline silicon plate (thickness 0.7 mm, resistivity 0.001 Ω·cm). **(a)** Raw single-crystalline silicon wafer and **(b)** after thermal oxidation for 3 min.

Figure S1 shows the SEM image of a single-crystalline silicon plate (thickness 0.7 mm, resistivity 0.001 Ω·cm). There is no grain or grain boundary on the surface of the Si plate even at a magnification of 90,000 times, which is consistent with the measured XRD results, indicating that the SiO_2 film generated by thermal oxidation was amorphous.

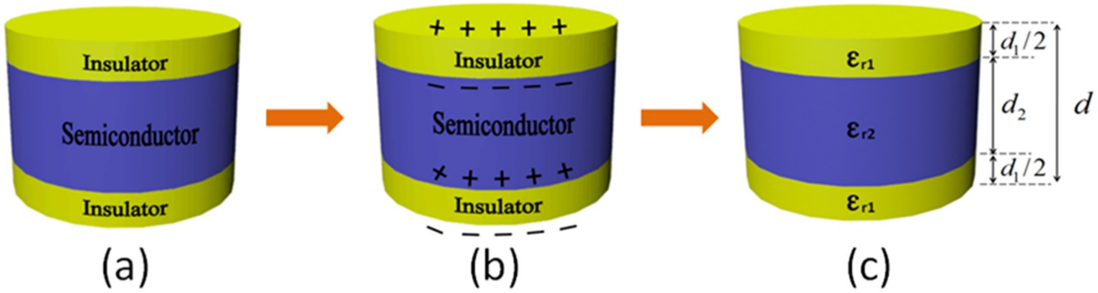

Figure S2. Schematic of colossal permittivity based on the thermal oxidation for 3 min single-crystalline silicon plates (thickness 0.7 mm, resistivity 0.001 Ω·cm) in O_2 atmosphere. **(a)** Insulator/semiconductor/insulator sandwich structure. **(b)** Sandwich structure applied by an external electrical field. **(c)** Dielectric constant and thickness of each part in the sandwich structure. ε_{r1} (ε_{r2}) and d_1 (d_2) are dielectric constant and thickness of the insulator (semiconductor), respectively. The total thickness of the sandwich structure is labeled as d, which is the sum of d_1 and d_2 ($d_2 >> d_1$).

Figure S2 shows the schematic of colossal permittivity based on the thermal oxidation for 3 min single-crystalline silicon plates (thickness 0.7 mm, resistivity 0.001 Ω·cm) in O_2 atmosphere.

According to the theory of capacitor in series, the overall capacitor 'C' of the sandwich structure is expressed as:

$$\frac{1}{C}=\frac{1}{C_1}+\frac{1}{C_2}+\frac{1}{C_1}=\frac{2}{C_1}+\frac{1}{C_2} \tag{1}$$

where C_1 and C_2 are the capacitors of the insulator and the semiconductor layer, respectively. Both the insulator and the semiconductor layer are reckoned as a plate capacitor, and the total capacitor 'C' of the sandwich structure, the capacitor 'C_1' of the insulator layer, and the capacitor 'C_2' of the semiconductor are:

$$C=\varepsilon_0\,\varepsilon_r s/d;\ C_1=2\varepsilon_0\,\varepsilon_{r1}s/d_1;\ C_2=\varepsilon_0\,\varepsilon_{r2}s/d_2 \tag{2}$$

respectively. Here, ε_0 is the absolute dielectric constant and s is the surface area of the sandwich structure; ε_{r1} (ε_{r2}) and d_1 (d_2) are dielectric constant and thickness of the insulator (semiconductor), respectively. The total thickness of the sandwich structure is labeled as d, which is the sum of d_1 and d_2 (d_2>>d_1, $d \approx d_2$). The dielectric constant ε_r of the sandwich structure is derived as

$$\varepsilon_r=\frac{d_2\varepsilon_{r1}\varepsilon_{r2}}{d_1\varepsilon_{r2}+d_2\varepsilon_{r1}}=\frac{\varepsilon_{r2}}{\frac{d_1\varepsilon_{r2}}{d_2\varepsilon_{r1}}+1} \tag{3}$$

Here, Equation (3) can be expressed as three sub-formulas:

$$\varepsilon_r=\begin{cases}\frac{\varepsilon_{r2}}{2}; & \frac{d_1\varepsilon_{r2}}{d_2\varepsilon_{r1}}=1\\ \varepsilon_{r2}; & \frac{d_1\varepsilon_{r2}}{d_2\varepsilon_{r1}}\ll 1\\ \frac{d_2}{d_1}\varepsilon_{r1}; & \frac{d_1\varepsilon_{r2}}{d_2\varepsilon_{r1}}\gg 1\end{cases} \tag{4}$$

The above three sub-formulas show that ε_r of the sandwich structure is dependent of ε_{r2} (dielectric constant of the semiconductor) and d_2/d_1 (the ratio of the thickness between the semiconductor and the insulator). Only if both ε_{r2} and d_2/d_1 are large enough, a relative great valve of ε_r can be obtained.

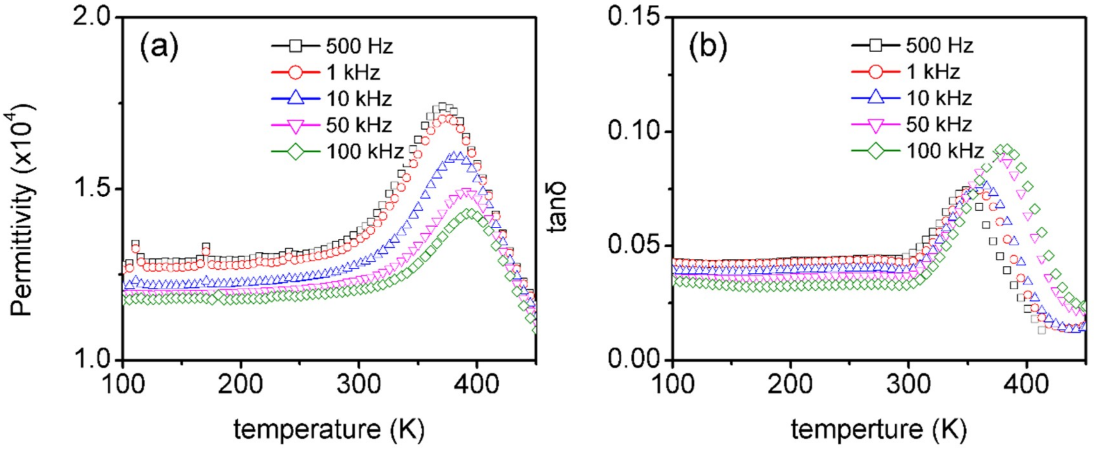

(**a**) (**b**)

Figure S3. Temperature dependence of the dielectric properties at difference frequency (500 Hz, 1 kHz, 10 kHz, 50 kHz and 100 kHz) were measured from 100 to 450 K. Dielectric permittivity **(a)** and dielectric loss **(b)** of the single-crystalline silicon plate (thickness 0.7 mm, resistivity 0.001 Ω·cm) after thermal oxidation for 3 min.

Figure S3 shows temperature dependence of the dielectric properties at difference frequency. The measured sample was the single-crystalline silicon plate (thickness 0.7 mm, resistivity 0.001 Ω·cm) after thermal oxidation for 3 min. The temperature range was from 100 to 450 K, which was limited

by our experiment condition. It is of significance that both colossal permittivity (CP) and low dielectric loss of the thermal oxidized single-crystalline silicon plate are almost independent of temperature over a wide temperature range, from 100 K up to 300 K. The relaxation peak at about 350 K may be due to the effect of carrier hopping at high temperature, which is similar to those of nano-TiO_2 bulks, Ti + Nb co-doped TiO_2 [2,3] and $Ce_{1-x}Gd_xO_{2-\delta}$ ceramics [4].

References

1. Deal, B.E.; Grove, A.S. General Relationship for the Thermal Oxidation of Silicon. *J. Appl. Phys.* **1965**, *36*, 3770–3778.
2. Zhang, L.D.; Zhang, H.F.; Wang, G.Z. Dielectric behaviour of nano-TiO_2 bulks. *Phys. Status Solidi A* **1996,** *157*, 483–491.
3. Guo, B.; Liu, P.; Cui, X. Colossal permittivity and dielectric relaxations in Tl + Nb co-doped TiO_2, ceramics. *Ceram. Int.* **2018**, *44*, 12137.
4. Zhu, G.B.; Guo, Y.M. Dielectric behavior of $Ce_{1-x}Gd_xO_{2-\delta}$ (0 <= x <= 0.1) ceramics prepared by sol-gel method. *J. Alloy Compd.* **2019,** *785*, 590–597.

© 2019 by the authors. Submitted for possible open access publication under the terms and conditions of the Creative Commons Attribution (CC BY) license (http://creativecommons.org/licenses/by/4.0/).
